# Methods to account for uncertainties in exposure assessment in studies of environmental exposures

**DOI:** 10.1186/s12940-019-0468-4

**Published:** 2019-04-08

**Authors:** You Wu, F. Owen Hoffman, A. Iulian Apostoaei, Deukwoo Kwon, Brian A. Thomas, Racquel Glass, Lydia B. Zablotska

**Affiliations:** 1 Department of Epidemiology and Biostatistics, University of California, San Francisco, 550 16th Street, 2nd floor, Box 0560, San Francisco, CA 94143 USA; 2 Oak Ridge Center for Risk Analysis, Inc., 102 Donner Drive, Oak Ridge, TN USA; 3 Sylvester Comprehensive Cancer Center, University of Miami, 1475 NW 12th Avenue, Miami, FL USA; 4 Center for Design and Analysis, Amgen, Inc., 1 Amgen Center Dr., Thousand Oaks, CA 91320 USA

**Keywords:** Environmental exposure, Radiation exposure, Risk assessment, Uncertainty, Measurement error, Regression calibration, Simulation-extrapolation, Monte Carlo maximum likelihood, Bayesian model averaging

## Abstract

**Background:**

Accurate exposure estimation in environmental epidemiological studies is crucial for health risk assessment. Failure to account for uncertainties in exposure estimation could lead to biased results in exposure-response analyses. Assessment of the effects of uncertainties in exposure estimation on risk estimates received a lot of attention in radiation epidemiology and in several studies of diet and air pollution. The objective of this narrative review is to examine the commonly used statistical approaches to account for exposure estimation errors in risk analyses and to suggest how each could be applied in environmental epidemiological studies.

**Main text:**

We review two main error types in estimating exposures in epidemiological studies: shared and unshared errors and their subtypes. We describe the four main statistical approaches to adjust for exposure estimation uncertainties (regression calibration, simulation-extrapolation, Monte Carlo maximum likelihood and Bayesian model averaging) along with examples to give readers better understanding of their advantages and limitations. We also explain the advantages of using a 2-dimensional Monte-Carlo (2DMC) simulation method to quantify the effect of uncertainties in exposure estimates using full-likelihood methods. For exposures that are estimated independently between subjects and are more likely to introduce unshared errors, regression calibration and SIMEX methods are able to adequately account for exposure uncertainties in risk analyses. When an uncalibrated measuring device is used or estimation parameters with uncertain mean values are applied to a group of people, shared errors could potentially be large. In this case, Monte Carlo maximum likelihood and Bayesian model averaging methods based on estimates of exposure from the 2DMC simulations would work well. The majority of reviewed studies show relatively moderate changes (within 100%) in risk estimates after accounting for uncertainties in exposure estimates, except for the two studies which doubled/tripled naïve estimates.

**Conclusions:**

In this paper, we demonstrate various statistical methods to account for uncertain exposure estimates in risk analyses. The differences in the results of various adjustment methods could be due to various error structures in datasets and whether or not a proper statistical method was applied. Epidemiological studies of environmental exposures should include exposure-response analyses accounting for uncertainties in exposure estimates.

## Background

Environmental epidemiological studies are designed to examine the impact of potentially toxic exposures on the health of occupationally exposed workers and members of the public [1]. These studies provide valuable information to public health authorities, especially with regard to health risks of hazardous environmental exposures [2]. The exposure estimate in such studies is usually a complex system which describes physical, chemical or biological characteristics of hazardous substances along with their transport mechanisms in the general environment or workplace over time and space. In addition, the role of individuals needs to be considered in exposure estimation to determine the mechanism of uptake as well as the amount of uptake of toxic substances by the human body. Such complex processes lead to formidable challenges in exposure estimation as well as make the issue of estimation error unavoidable.

In the past two years, about 2000 papers have been published which included some kind of risk analysis of the effects of environmental exposures. However, only 39 of these publications mentioned that ‘measurement error’ or ‘uncertainties’ may exist in exposure assessments. A smaller amount of these publications (15) have assessed the effect of measurement and/or estimation errors on risk estimates. Failure to account for uncertainties in exposure estimation may lead to biased results and undue confidence in their accuracy in the subsequent exposure-response analyses. As a result, inaccurate information about the risks of exposures may be distributed to other scientists, the public and to decision makers. The three main effects of performing an exposure-response analysis based on the error-prone exposure estimates are: (a) biased estimation of exposure-response parameters, (b) reduction of statistical power, and (c) hidden true exposure-response features (e.g., true exposure-response is distributed with a certain cyclic variation pattern such as sinusoidal trend, however, this feature may be masked if exposure is estimated with errors) [3].

Ionizing radiation is a known and well-studied carcinogen [4]. The effects of potential errors in exposure estimation on the radiation dose-response has been debated in radiation epidemiology for a number of years [5]. The process of estimation of radiation doses is usually subject to various sources of uncertainties [6, 7]. Little et al. (2015), Land et al. (2015) and others used a variety of statistical methods to examine the impact of uncertainties in individual dose estimates on risk estimates in different populations exposed to ionizing radiation [8, 9]. However, the topic of uncertainties in exposure estimation is not commonly considered for other exposure types in environmental epidemiological studies. The goal of this paper is to introduce and review various error types in exposure estimation as well as the statistical approaches to account for exposure estimation errors in risk analyses. The approaches reviewed are regression calibration, simulation-extrapolation, Monte Carlo maximum likelihood and Bayesian model averaging. We will summarize their advantages and limitations as well as provide suggestions for application of each method to other relevant scenarios in environmental epidemiological studies.

## Main text

**Fig. 1:**
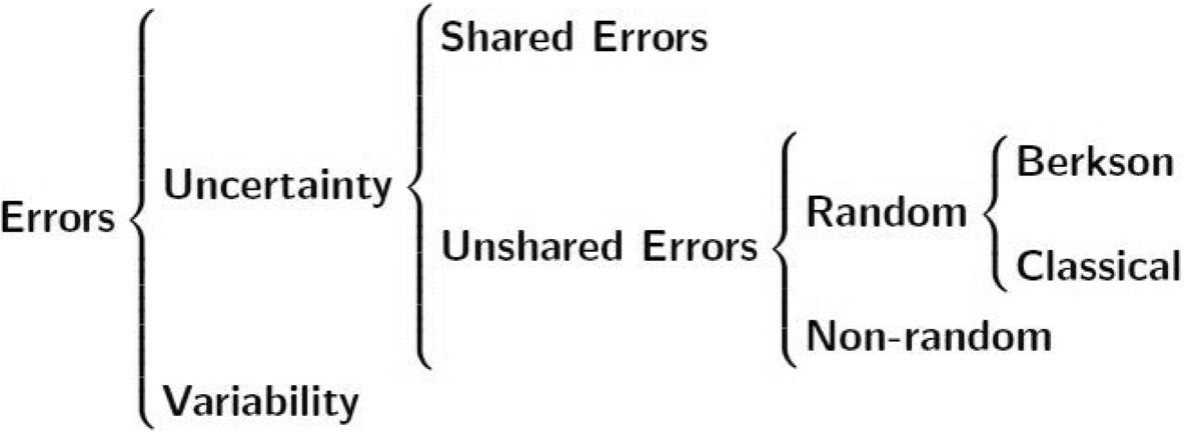
A diagram of general error types in exposure measurements

Exposure uncertainties could be evaluated based on investigator’s knowledge about distribution of each parameter required to estimate individual exposure values [10]. The various sources of exposure estimation errors may result in different types of errors which would require different approaches to minimize their effects on risk estimates. In this section, different error types in exposure estimation, statistical methods to account for exposure estimation errors and representative studies that applied these methods are reviewed. Figure 1 shows a diagram of various types of exposure estimation errors (adapted from [7]). Potential sources and relevant examples of each type of error are described in Table 1. Representative studies in radiation epidemiology and other environmental epidemiological fields are listed in Tables 2 and 3, respectively.

**Table 1:** Possible sources and examples for each error type

Error Types	Possible Sources	Examples
Variability	Differences in individual’s location, exposure or behavior, randomness, etc.	Individual-specific exposure estimates differ with distances from the pollutant source [12]; Exposure to pesticides or bacteria may vary by season [12]; Different patterns of food intake may result in different exposures across individuals [12].
Uncertainty	Lack of knowledge in specifying exposure pathways, simplified model assumptions, failure to account for possible correlations between variables, etc.	There is uncertainty in the level of exposure to insecticide sprays due to the unknown exposure pathway (inhalation, dermal contamination or both) [61]; There is uncertainty when calculating one’s inhalation rate because of the failure to account for the dependency of body weight and breathing volume [61]; There is uncertainty in the estimated room air concentration because of the unknown release rate of the chemical. [61].
Shared Error	Incomplete knowledge about the parameters that affect the exposure measurements of group.	Inaccurate estimations of the ground deposition of certain contaminants may affect the estimation of exposure for all people who live in the same area [18]; Errors from an uncertain of a biased measuring device when it is used to a group of people [54].
Unshared Error	Lack of knowledge about the parameters that vary randomly between subjects.	See the examples for classical error, Berkson error and unshared non-random error.
Classical Error	Imprecise measuring device, repeated measurements that vary around the true value, etc.	Using the replicated urinary nitrogen as a measured biomarker to investigate the true long-term dietary protein intake, etc. [3, 44]
Berkson Error	The same exposure value is assigned to a group with similar characteristics.	Air quality records collected by a monitoring station are assigned to all subjects in the study as estimates of true individual exposure to pollutants [62]; When job-exposure-matrix is used to estimate the individual exposure in occupational epidemiological studies, same exposure estimate is assigned to the groups of people with same occupation code [17, 63].
Unshared Non-random Error	Imprecise knowledge in individual specific parameters.	Errors in personal residence history records [7]; Errors in personal consumption rates of contaminated foods [7].

**Table 2:** Examples of results from radiation epidemiological studies with and without adjusting for exposure estimation errors

Studies	Exposure	Outcome	Error-correction Method	Measures of Association	Unadjusted risk effect with 95% confidence interval	Adjusted risk effect with 95% confidence interval	Percent change after adjustment
Little et al. (2015) [9]	131-I thyroid dose	Thyroid cancer	Regression Calibration	Excess odds ratio/Gy	1.51 (0.53, 3.86)	1.31 (0.47, 3.31)	−13%
			MCML	Excess odds ratio/Gy	1.51 (0.53, 3.86)	1.48 (0.53, 3.87)	− 2%
			BMA	Excess odds ratio/Gy	1.51 (0.53, 3.86)	1.16 (0.20,4.32) **	−23%
Land et al. (2015) [8]	External thyroid dose	Thyroid nodules	BMA	Excess relative risk/Gy	4.16 (0.54, 7.77)	1.47 (4.1E-05,3.74) **	−64%
	Internal thyroid dose		BMA	Excess relative risk/Gy	0.88 (0.24, 1.52)	3.59 (0.11, 9.73) **	+ 308%
Kesminiene et al. (2008) [64]	Gamma-ray bone marrow dose	Hematological malignancies	MCML	Relative risk at 100 mGy	0.60 (− 0.02, 2.35)*	0.60 (− 0.01 2.58)*	0%
Stayner et al. (2007) [21]	Gamma-ray whole-body dose	All cancer mortality (excluding leukemia)	MCML	Excess relative risk/Sv	5.38 (0.54, 12.58)*	4.82 (0.41, 13.31)*	−10.4%
Allodji et al. (2015) [50]	Gamma-ray colon dose	Solid cancer deaths	Regression Calibration	Excess relative risk/Gy	0.43 (0.35, 0.51)	0.46 (0.38, 0.55)	+ 6.7%
			SIMEX	Excess relative risk/Gy	0.43 (0.35, 0.51)	0.60 (0.50, 0.69)	+ 38.4%
	Gamma-ray bone marrow dose	Leukemia deaths	Regression Calibration	Excess relative risk/Gy	3.86 (2.70, 5.02)	4.14 (2.90, 5.39)	+ 7.3%
			SIMEX	Excess relative risk/Gy	3.86 (2.70, 5.02)	4.62 (3.30, 5.93)	+ 19.6%

*90% Confidence interval

**95% Bayesian credible interval

**Table 3:** Examples of results of non-radiation environmental epidemiological studies with and without adjusting for exposure estimation errors

Studies	Exposure	Outcome	Error-correction Method	Measures of Association	Unadjusted risk effect with 95% confidence interval	Adjusted risk effect with 95% confidence interval	Percent change after adjustment
Kumar (2016) [48]	PM2.5 (μg/m3)*	Birth weight (g)	SIMEX	Linear regression coefficient	−1.01 (−2.017, 0.003)	−0.98 (−1.689, − 0.263)	+ 3%
	PM10 (μg/m3)**	Birth weight (g)	SIMEX	Linear regression coefficient	−1.01 (−2.017, 0.003)	−0.98 (−1.689, − 0.263)	+ 0.1%
Wang and Song (2016) [56]	% Energy from protein	Breast cancer	Regression Calibration	Hazard ratio	−0.15 (−1.79,1.49)	0.53 (−1.74, 0.72)	− 248%
Keogh and White (2014) [39]	Log fiber intake (gram/day)	Colorectal cancer	Regression Calibration	Log odds ratio	− 0.19 (− 0.33, − 0.06)	− 0.29 (− 0.49, − 0.09)	−48.2%
Beulens et al. (2007) [65]	Alcohol consumption	Total myocardial infarction	Regression Calibration	Hazard ratio	0.85 (0.78, 0.92)	0.68 (0.46, 1.00)	−20%
	Alcohol consumption	Total death	Regression Calibration	Hazard ratio	0.96 (0.91, 1.02)	0.94 (0.75, 1.18)	−2%
	Alcohol consumption	Total stroke	Regression Calibration	Hazard ratio	1.03 (0.92, 1.15)	1.04 (0.72, 1.51)	+ 0.9%
Molina-Montes et al. (2012) [66]	Energy-adjusted magnesium intake	Pancreatic cancer	Regression Calibration	Hazard ratio	0.98 (0.86, 1.11)	0.68 (0.46, 1.00)	+ 2%
	Energy-adjusted iron intake		Regression Calibration	Hazard ratio	0.97 (0.91, 1.03)	0.94 (0.75, 1.18)	−4%
	Energy-adjusted heme-iron intake		Regression Calibration	Hazard ratio	1.01 (0.90, 1.12)	1.04 (0.72, 1.51)	+ 7%
Beydoun et al. (2007) [67]	n-3 C18 polyunsaturated fatty acids	Cognitive functioning (measured by Word Fluency Test)	Regression Calibration	Odds ratio	1.01 (0.92, 1.12)	1.22 (0.90, 1.64)	+ 20.8%
	n-3 C18 polyunsaturated fatty acids	Cognitive functioning (measured by Word Fluency Test)	SIMEX	Odds ratio	1.01 (0.92, 1.12)	1.15 (0.96,1.39)	+ 13.9%

*particulate matter (PM) ≤ 2.5 μm in diameter

**particulate matter (PM) ≤ 10 μm in diameter

### Error types

#### Uncertainty vs. variability

“Uncertainty” is sometimes defined as all possible sources that challenge the study’s validity (e.g., [11]). In such cases, variability is considered a special type of uncertainty. However, the U.S. Environmental Protection Agency (EPA) has suggested that researchers should follow the definitions of uncertainty and variability recommended by National Research Council (NRC), which distinguish the natures of these two error types ([12, 13]). According to the NRC (1994) definition, “uncertainty” is defined as a lack of precise knowledge that is presented during exposure assessment procedures and is due to absence of or imprecise measurements, observations or information pertinent to the assessment question. However, variability in exposure reflects the inherent heterogeneity of the exposure across individuals. Inter-individual variability of the unknown true exposure or dose will still exist due to randomness even if all other identified exposure characteristics (such as sex, age, life-style, location of residence, diet, job identifiers, etc.) are identical across a set of individuals [5, 7, 12].

#### Shared errors vs. unshared errors

Shared uncertainties are introduced when there is incomplete knowledge about model parameters that are used to estimate the exposure of a subgroup of individuals in a cohort. As a limit, uncertainties can be shared among parameters that apply to all members of the cohort (i.e., the subgroup can be equal to the entire cohort). The true values of these parameters are unknown but fixed (i.e., not varying on an individual-by-individual basis). The errors in these parameters lead to systematic errors in exposure estimates of all subgroup members [7]. In epidemiological studies, shared error (systematic error) refers to bias. Unshared errors, which usually refer to random errors, are the uncertainties that arise from parameters that vary independently between subjects. An unshared error could be random, which is usually classified into two types: classical error and Berkson error. It also could be non-random (e.g., errors in personal residence history records) because the true residence information is fixed to a specific individual [7].

#### Classical error vs. Berkson error

Both classical error and Berkson error are types of unshared random error. Classical error stems from an imprecise measuring device that is used to estimate individual exposure. It is also introduced by over-estimation of inter-individual random variability of true exposure. This error is most commonly defined as a situation when there are repeated measurements which vary around the unknown true value for each individual. Berkson errors are introduced when the same approximate exposure value (usually the arithmetic mean value for a group) is assigned to each member of a cohort sub-group who share similar exposure characteristics. The true exposure values for individuals in this group are unknown, but vary around the assigned value [14]. Examples of each error type are given in Table 1.

Suppose we are interested in an exposure *D* (e.g., radiation dose), and *D*^*tr*^ represents an unknown true value of the exposure, while *D*^*est*^ represents an estimated value of exposure *D*. In many studies, but especially when exposure refers to a radiation dose, the “measured” value is usually not directly used in the exposure-response analysis, and calibrations and calculations are applied to the “measured” value to obtain a final “estimated” exposure value. This “estimated” value will be used in the subsequent risk analyses. Thus, we prefer to use the term of “estimated exposure” rather than “measured exposure” in this paper in order to avoid misinterpretation. Using these notations, the classical error model is expressed as

$$ {D}^{est}={D}^{tr}+{U}_c $$

where *U*_*c*_ is a classical error term with *E*(*U*_*c*_| *D*^*tr*^) = 0 and the estimated exposure *D*^*est*^ is an unbiased estimate of the true exposure, that is, *E*(*D*^*est*^| *D*^*tr*^) = *D*^*tr*^. When the error term *U*_*c*_ has a constant variance, ${\sigma}_u^2$, *U*_*c*_ ∣ *D*^*tr*^ approximately follows a normal distribution [3], although other types of distributions may apply.

On the other hand, the Berkson error model could be expressed as

$$ {D}^{tr}={D}^{est}+{U}_b $$

where *E*(*U*_*b*_| *D*^*est*^) = 0, and *E*(*D*^*tr*^| *D*^*est*^) = *D*^*e*s*t*^.

For the studies with exposure measured independently between subjects, unshared errors are more likely to occur in exposure estimation. For example, when self-report values are used as individual exposures, almost all the uncertainties are from unshared components. In contrast, when a biased/uncalibrated measuring device is used or mathematical models to estimate exposure with uncertainty on mean values for the model parameters are applied for a group of people, shared errors are more likely to be introduced. For example, when a mathematical model is used to define the transport mechanism of a toxicant, shared uncertainties would be potentially large if this model is not well designed (i.e., it does not characterize the true transport features perfectly). Uncertainties introduced from this model will usually affect the entire cohort. In such cases, shared uncertainties could not be ignored in exposure estimates. Differentiation of classical error from Berkson error is relatively easy in practice. If the error-prone exposure is estimated uniquely to an individual, especially when some measurements during exposure estimation could be replicated, the errors should be considered classical. If a group of people are assigned the same value (usually the group average) of the error-prone exposure while the true exposure value is particular to an individual, errors are considered to be Berkson type [3, 15, 16].

**Fig. 2:**
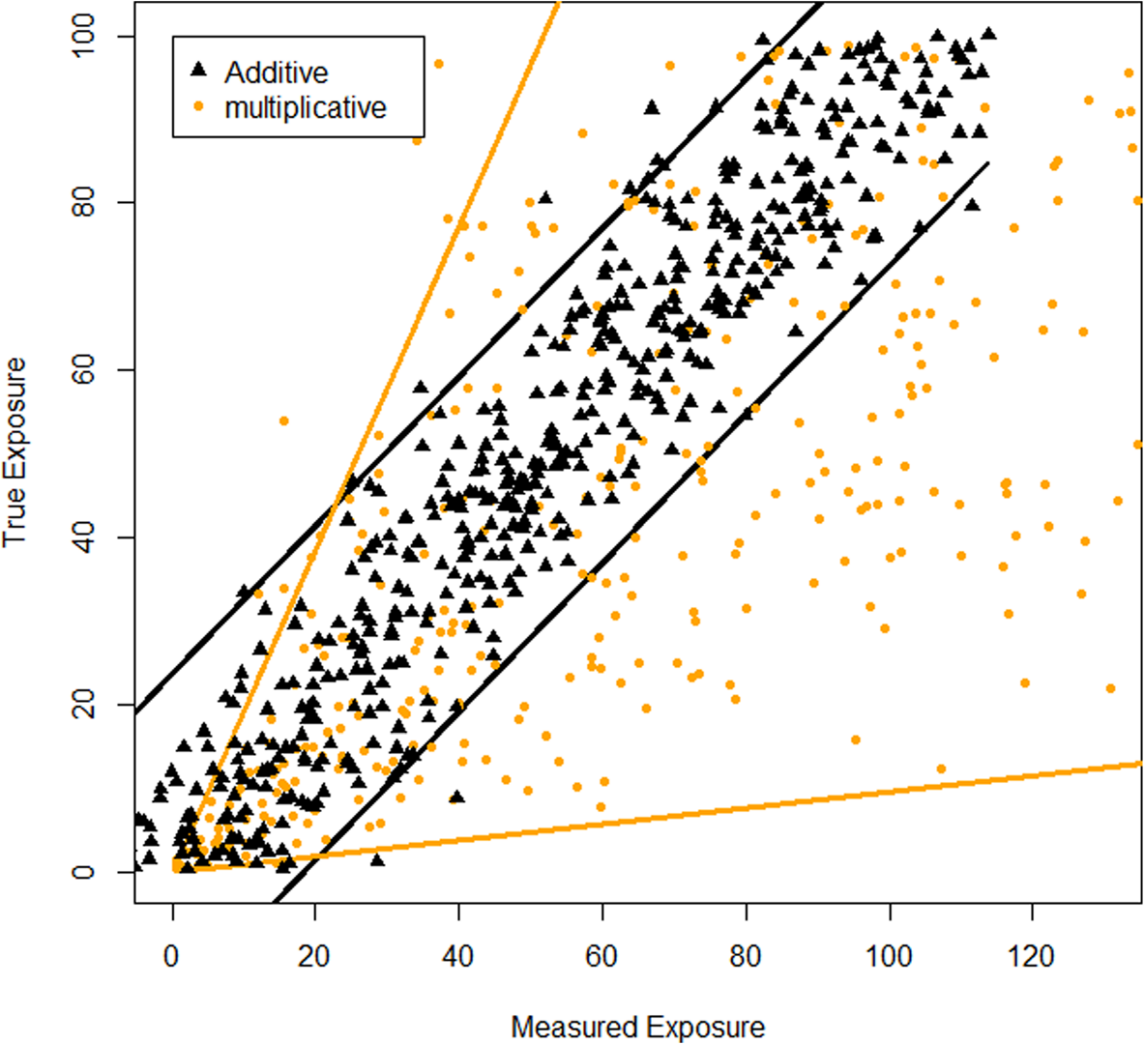
Measured exposure vs. true exposure assuming additive error model and multiplicative error model (adapted from [17])

Two types of error structure are usually considered in the analysis of exposure estimation error. They are described by a multiplicative error model or by an additive error model. The multiplicative error structure is considered when the spread of the true exposure given the estimated exposure increases proportionally to the estimated exposure values, while the additive error structure should be considered when the spread remains constant [17]. The true values of the exposure are unknown, but one can plot the average values of replicated exposure estimates per individual versus each of the replicated individual exposure estimates. When the plot (made on a linear scale) is in a “tube” shape, the error structure is most likely described by an additive error model, while a multiplicative model seems to be reasonable when the plot has a “trumpet” shape (Fig. 2) [17]. A “tube” shape of the plot displayed using a log scale indicates a multiplicative error.

### Use of a two-dimensional Monte Carlo method for estimation of exposures

In practice, the error structure of exposure estimation is usually complex and contains various types of errors, although one type usually predominates. In such cases, more advanced statistical methods are needed to account for the complex error structures in risk analyses. A Monte Carlo simulation procedure (i.e., repeated drawing of random samples from probability distributions of various exposure estimation parameters) could be used to generate multiple exposure estimates per individual (e.g., [8, 18, 19]). In this section, we introduce an exposure estimation approach called the two-dimensional Monte Carlo method (2DMC), which is an advanced approach compared to other forms of Monte Carlo methods widely used for quantitative uncertainty analysis in radiation dose reconstruction. By applying 2DMC in exposure estimation, information on both shared and unshared uncertainties are presented in the form of multiple alternative realizations of possibly true exposure estimate vectors. Each realization of a possibly true exposure estimate vector represents a set of different values of shared and unshared parameters. These multiple realizations of possibly true exposure estimate vectors allow researchers to use various statistical approaches to account for shared and unshared sources of exposure estimates of uncertainties in exposure-response analyses. This method allows researchers to use information about both shared and unshared uncertainties in exposure estimates in risk analyses. Although this method is time-consuming and challenging, it is often necessary for performing certain types of advanced statistical analyses of exposure-response accounting for errors in exposure estimates. The statistical methods that account for exposure estimation errors in exposure-response analyses are introduced in the later section, and are all described based on exposure estimates obtained by 2DMC. Although not all of them require that they be performed based on the 2DMC procedure, we use this setting for the convenience of comparison.

The 2DMC method is a simulation-based exposure reconstruction strategy that properly maintains the separation between shared uncertainties in exposure estimates among the entire cohort or the cohort’s subset, and the unshared, individual uncertainties. The concept of 2DMC is first mentioned in [20] while detailed implementation procedures were proposed by [7]. Although it had originally been proposed as a radiation dose reconstruction method, 2DMC could also be applied in other exposure scenarios in which the estimation procedure is complex and shared uncertainties are expected to be relatively large. By applying 2DMC, the parameters shared by cohort or subgroup members are fixed in the outer loop while the unshared parameters are simulated in the inner loop. Each run of the outer loop pass will generate a set of simulated exposure values for the *N* cohort members. For example, if the outer loop pass is run *M* times, it will result in a final estimated exposure *D*^*est*^ in a matrix form as below:

1 $$ {D}^{est}=\left(\begin{array}{ccc}{D}_{11}^{est}& \cdots & {D}_{1M}^{est}\\ {}\vdots & \ddots & \vdots \\ {}{D}_{N1}^{est}& \cdots & {D}_{NM}^{est}\end{array}\right) $$

where *N* is the sample size of the cohort. Thus, *M* sets of exposure are estimated for the entire cohort.

We use *W* to denote the full set of input data that is needed to determine the estimated exposure *D*^*est*^, where *W* does not represent a single variable but include all the variables needed in exposure estimation. Then, for example, in a dosimetry system developed to estimate radiation doses, *W* may include residence history, exposed age, intake of milk contaminated with radionuclides, etc. [21]. We use $f\left({D}_1^{tr},\dots, {D}_N^{tr}|W\right)$ to denote the joint distribution of true exposure for all cohort members, given all input data that is needed in exposure estimation. The aim of the exposure estimation procedure is thus to draw samples from $f\left({D}_1^{tr},\dots, {D}_N^{tr}|W\right)$ as potential exposure estimates. For 2DMC, where shared parameters are first fixed in the outer loop and the correlations among individuals are held, each estimated exposure vector ${D}_r^{est}\ \left(r=1,\dots, M\right)$ is sampled from the joint distribution $f\left({D}_1^{tr},\dots, {D}_N^{tr}|W\right)$ for all members of the cohort [21, 22]. Therefore, the estimated exposure matrix *D*^*est*^ in (1) is constructed by sampling $\left({D}_1^{est},\dots, {D}_N^{est}\right)$ for *M* times.

Each estimated set of exposures for the entire cohort $\left({D}_1^{est},\dots, {D}_N^{est}\right)$ based on 2DMC maintains the shared information among individuals and can possibly be the true exposure vector. When full-likelihood methods, such as the Monte Carlo Maximum Likelihood method and the Bayesian model averaging method, are applied to explore exposure-response relationship using the entire estimated sets of exposure, the overall effect of uncertainties in exposure estimates can be quantified [7]. Goodness of fit tests with respect to the cohort vector of individual exposure estimates and the cohort vector of individual disease incidence (or mortality) is used to distinguish between cohort exposure estimates that are plausible versus those that are not.

### Statistical methods to account for exposure estimate errors in exposure-response analyses

Below, we will review the four main statistical methods to account for effects of errors in exposure estimation on risk estimates. Each section presents a short description of the methods to estimate functions and associated variances, followed by examples and advantages and limitations. For more details, readers are referred to primary references. Examples of studies which successfully used these methods are provided in Tables 2 (radiation epidemiology studies) and 3 (studies of other environmental exposures).

### Regression calibration

Regression calibration is a replacement method [23] that substitutes the unobserved true exposure value *D*^*tr*^ by a calibration function E(*D*^*tr*^ ∣ *D*^*est*^) in the regression of the health outcome *Y* on true exposure *D*^*tr*^. The method could be easily applied to different types of data, including survival and binomial [24–30]. The general procedure of regression calibration can be summarized by the following three steps:

1. Estimate the calibration function *E*(*D*^*tr*^ ∣ *D*^*est*^);
2. Fit a regression of *Y* on *E*(*D*^*tr*^ ∣ *D*^*est*^) instead of the unobserved true exposure *D*^*tr*^;
3. Adjust the variance of the risk estimates to account for steps 1) and 2).

The method of estimation of calibration function E(*D*^*tr*^ ∣ *D*^*est*^) depends on the data sources. In situations where internal validation data or data on unbiased instrumental variables are available, the calibration function could be directly estimated by the regression of *D*^*tr*^ on *D*^*est*^ or by the regression of an unbiased instrumental variable on the estimated exposure [3].

When repeated estimates of exposure are available, the calibrated function could be estimated by the so-called linear approximation [3]. Suppose we have *M* replicates of exposure estimate for *i*^*th*^ individual $\left({D}_{i1}^{est},\dots, {D}_{iM}^{est}\ \right)$ and consider an additive classical error model: *D*^*est*^ = *D*^*tr*^ + *U*. The variance of error term *U* is then estimated by

2 $$ {\widehat{\sigma}}_u^2=\frac{\sum_{i=1}^N{\sum}_{j=1}^M{\left({D}_{ij}^{est}-{\overline{D}}_{i.}^{est}\right)}^2}{N\left(M-1\right)}\kern0.5em $$

where ${\overline{D}}_{i.}^{est}$ is the mean of *M* replicates for *i*^*th*^ individual. The best linear approximation to *D*^*tr*^ given *D*^*est*^ is given by

3 $$ E\left({D}^{tr}|{D}^{est}\right)\approx {\mu}_T+\frac{\sigma_T^2}{\sigma_T^2+\frac{\sigma_u^2}{M}}\left({\overline{D}}^{est}-{\mu}_{est}\right)\kern0.5em $$

where *μ*_*T*_ and *μ*_*est*_ are the means of *D*^*tr*^ and *D*^*est*^, respectively. Both of these variables could be estimated by the overall sample average $\frac{\sum_{i=1}^N{\overline{D}}_{i.}^{est}}{N}$, and the variance of the true exposure ${\sigma}_T^2$ is estimated by

4 $$ {\widehat{\sigma}}_T^2=\frac{M{\sum}_{i=1}^N{\left({\overline{D}}_{i.}^{est}-{\mu}_{est}\right)}^2-\left(N-1\right){\sigma}_u^2}{\left(N-1\right)M} $$

The formulas (2)–(4) above are based on the simple case which only considers a relationship between a single exposure *D* and outcome *Y* in a risk model. When other covariates *X* (usually assumed to be estimated without errors, e.g., age, gender, etc.) are included in the risk model, the calibration function would change to *E*(*D*^*tr*^| *D*^*est*^,  *X*). A matrix form of the linear approximation of *E*(*D*^*tr*^| *D*^*est*^,  *X*) could be found in [3]. For a multiplicative error model, the log transformation is used to convert it to an additive one. The method introduced above can then be directly applied to the log-transformed data. Statistical software such as Stata [31] could be used to calculate the adjusted standard error as well as the confidence interval. Although other methods are available to adjust the variance (see [3]), bootstrap is recommended for large data sets based on the speed of computations [32].

The regression calibration method has been used in several radiation epidemiological studies [9, 23, 33–36]. For example, a multiplicative error model was considered for estimated thyroid doses in studies of those exposed to the Chornobyl (Chernobyl) accident [9, 15, 34, 37]. By assuming that the error term was log-normally distributed, the calibration function *E*(*D*^*tr*^| *D*^*est*^) was obtained based on the conditional distribution of *f*(*D*^*tr*^| *D*^*est*^), which also follows a log-normal distribution. In analyses of Chornobyl data, risk analyses using regression calibration method to adjust for uncertainties in doses, had estimated excess odds ratios which were 7–11% higher in the Ukrainian cohort [34] and 13% higher in the Belarusian cohort [9] compared to conventional analyses without accounting for dose uncertainties.

Regression calibration method is also widely used in nutritional studies. A recent systematic review of measurement error-correction approaches in nutritional epidemiology, showed that 71 of 76 studies adjusted for exposure measurement errors by regression calibration method [38]. Nutrient intake measurements frequently have errors because they are usually assessed based on self-reported food frequency questionnaires (FFQ) [39, 40]. To apply a regression calibration method to adjust for the measurement errors in FFQ, researchers typically collect additional data for a reference variable in a subset of the population from the main study. The reference variable is usually measured by multiple 24-h dietary recalls, or some biomarkers, such as urinary nitrogen for protein intake [40–44]. Regression of this reference variable on dietary variable from the FFQ is treated as an estimate of the calibration function *E*(*D*^*tr*^ ∣ *D*^*est*^). Table 3 presents examples of studies that used regression calibration to adjust for exposure estimation errors.

### Simulation-extrapolation

The simulation-extrapolation (SIMEX) method is a simulation-based method that is implemented in two steps: a simulation step and an extrapolation step [45]. The simulation step seeks to explore the relationship between errors in exposure estimation and an estimator of interest. Based on this relationship, the error-free estimate of risk parameter is obtained by setting the variance of the error term to zero in the extrapolation step. In this case, the “error-free” estimate here does not imply a perfect estimate of the risk parameter but a parameter estimator. A log-transformation could also be applied to generate an additive error when a multiplicative error model is considered [3].

In the simulation step, a set of pre-selected parameters (*ξ*_1_,  … , *ξ*_*T*_), such that 0 ≤ *ξ*_1_ < *ξ*_2_ <  …  < *ξ*_*T*_ are used as the scale factors to construct pseudo-errors. A “contaminated” exposure data set (i.e., the data set to which extra errors are manually added) could be generated for each scale factor *ξ*_*t*_:

5 $$ {D}_{(t),i}^{est,\ast }={D}_i^{est}+\sqrt{\xi_t}\ {U}_i $$

where *i* = 1, … , *N*; *t* = 1, … , *T*; *U*_*i*_ is sampled from $N\left(0,{\sigma}_u^2\right)$ and ${\sigma}_u^2$ could be estimated using repeated data as eq. (2). Based on the “contaminated” data $\left({Y}_i,{D}_{(t),i}^{est,\ast}\right)$, a naïve parameter $\widehat{\beta}\left({\xi}_t\right)$ estimate could be obtained by fitting a regression model.

After the simulation step, the risk parameter estimate is obtained $\widehat{\beta}\left({\xi}_t\right)$ for each pre-selected scale factor *ξ*_*t*_, where $\widehat{\beta}\left({\xi}_t\right)$ could be treated as a function of *ξ*_*t*_. It is assumed that an extrapolation function, *G*(∙), is used to capture the relationship between the risk parameter estimate $\widehat{\beta}\left({\xi}_t\right)$ and the scale factor *ξ*_*t*_, that is, $\widehat{\beta}\left({\xi}_t\right)=G\left({\xi}_t;\gamma \right)$, where *γ* is the parameter in function *G*(∙). The extrapolation step is then summarized as follows:

1) Estimate the parameter *γ* in the extrapolant function *G*(*ξ*_*t*_; *γ*).

2) Obtain the SIMEX estimate for the risk parameter: ${\widehat{\beta}}_{SIMEX}=G\left({\xi}_t=-1;\widehat{\gamma}\right)$.

During the extrapolation step, it is important to decide how to choose the extrapolation function *G*(∙). Cook and Stefanski [45] suggested three different extrapolation functions which include a linear extrapolation *G*(*ξ*; *γ*) = *γ*_1_ + *γ*_2_*ξ*, a linear quadratic extrapolation *G*(*ξ*; *γ*) = *γ*_1_ + *γ*_2_*ξ* + *γ*_2_*ξ*^2^, and a nonlinear extrapolation function (also called the rational linear extrapolant) $G\left(\xi; \gamma \right)={\gamma}_1+\frac{\gamma_2}{\gamma_3+\xi }$. These extrapolants provide a relatively good approximation for any particular estimator.

The estimate of the standard error of the SIMEX estimator could be obtained via a bootstrap procedure, a Jackknife procedure [46], or a sandwich estimator [3]. SIMEX estimator with the estimated standard error could be obtained using statistical software such as Stata [31] or the R package “*simex*” [47].

SIMEX or extended SIMEX have been applied in some air pollution studies to adjust for errors in exposure estimates (e.g., [48, 49]). For example, a recent study of exposures to particulate matter (PM) estimated individual exposures using data from multiple monitoring stations within a certain area, which could potentially introduce some errors. After adjusting for exposure estimation errors by extended SIMEX, the estimated effect of PM < 2.5 μm in diameter (PM_2.5_) on birth weight increased by 56.7% in Alexeeff et al. [49] compared to analyses without adjustments for errors in exposure estimation. A radiation epidemiological study exploring a relationship between individual colon dose from gamma radiation and solid cancer deaths [50] reported that the estimated excess relative risk per gray (ERR/Gy) increased by 38.4% after accounting for dose uncertainties by SIMEX, compared to an increase of 6.7% after adjustment by regression calibration. Similar increases in risk estimates were reported in a study of effects of bone marrow doses on the risk of death from leukemia in survivors of atomic bombings in Japan [50]. After adjusting for dose uncertainties by SIMEX, the estimated ERR/Gy increased by 19.6%, compared to an increase of 7.3% after adjustment using regression calibration method (see Table 3 for details).

### Monte Carlo Maximum Likelihood

The estimated exposure matrix *D*^*est*^ from equation (1) from the 2DMC dosimetry system can be treated as a sample drawn from the conditional distribution of true exposure given the input data *f*(*D*^*tr*^| *W*). Because *W* represents the observed values of all the data that are used to determine the exposure estimates, we could estimate an observed likelihood *f*(*Y*| *W*; *α*, *β*) in the exposure-response analysis [21], where *α* and *β* are the parameters of covariates and exposure, respectively. The basic idea behind the Monte Carlo Maximum Likelihood (MCML) method is to obtain a maximum likelihood estimate of the risk parameter *β* based on the observed likelihood *f*(*Y*| *W*; *α*, *β*) [21, 22] from multiple dose vectors. The observed likelihood can be expressed as

6 $$ f\left(Y|W;\alpha, \beta \right)={E}_{D^{tr}\mid W}\left[f\left(Y|{D}^{tr};\alpha, \beta \right)\right] $$

where ${E}_{D^{tr}\mid W}\left[\bullet \right]$ indicates the expectation under the conditional distribution of the true exposure *D*^*tr*^ given the full set of input data *W* and *f*(*Y*| *D*^*tr*^; *α*, *β*) represents the exposure-response model that describes the relationship between response and true exposure value [21]. Since the estimated exposure *D*^*est*^ can be treated as multiple samples drawn from the conditional distribution *f*(*D*^*tr*^| *W*), the observed likelihood can be estimated by averagingdexposure vectors:

7 $$ f\left(Y|W;\alpha, \beta \right)=\frac{1}{M}\sum \limits_{r=1}^M{f}_{Y\mid {D}^{tr}}\left(Y|{D}_r^{est};\alpha, \beta \right) $$

where ${D}_r^{est}\ \left(r=1,\dots, M\right)$ is the estimated exposure vector for the entire cohort. For a set of pre-selected values of *β*, [*β*_1_,  … , *β*_*K*_], the profile likelihood of *β* is expressed as

$$ L\left({\beta}_k\right)={\mathit{\max}}_{\alpha }f\left(Y|W;\alpha, {\beta}_k\right) $$

8 $$ \kern9.5em ={\mathit{\max}}_{\alpha}\left\{\frac{1}{M}\sum \limits_{r=1}^M{f}_{Y\mid {D}^{tr}}\left(Y|{D}_r^{est};\alpha, {\beta}_k\right)\right\} $$

Then the maximum likelihood estimate of *β* is the *β* value that maximizes the profile likelihood: ${\widehat{\beta}}_{MLE}={argmax}_{\beta}\left[L\left({\beta}_k\right)\right]$. The likelihood ratio test statistic, $-2\ln \left[L\left(\beta \right)\right]+2\ln \left[L\left({\widehat{\beta}}_{MLE}\right)\right]$, has an asymptotic *χ*^2^ distribution with one degree of freedom [21] and can be used to estimate a confidence interval.

For a complex dosimetry system, simple (unweighted) average might not produce precise values for point estimate and confidence interval for *β* since only a few exposure vectors will have reasonable goodness-of-fit to the response. In such cases, it would be better to implement MCML based on weighted average of profile likelihood function with respect to the goodness-of-fit measure such as Akaike information criterion (AIC) and Bayesian information criterion (BIC).

The MCML method has been used in many radiation studies. For example, in the 15-country study of cancer risks of nuclear workers [21], a time-period- and facility-specific bias factor was introduced to calculate possible true doses. The uncertainties in this bias factor were shared across all individuals who worked in the same facility during the specified time period. In analyses with MCML, the estimated ERR per unite dose (ERR/Sievert (Sv)) was reduced by 10.4% compared to the unadjusted estimate (see Table 2).

### Bayesian model averaging

Kwon et al. (2016) proposed a Bayesian model averaging (BMA) method to account for uncertainties in exposure estimates [51]. This method uses a data augmentation approach to the multiple estimated exposure vectors obtained from 2DMC by introducing an exposure vector selection parameter, say *γ* (*γ* = 1,  … , *M*). Bayesian inference could be treated as a learning process from the opinion of the unknown parameters (i.e., prior distribution) and the data at hand (i.e., likelihood). By first sampling one value of the vector selection parameter *γ* from its prior distribution, one of *M* exposure vectors will be selected as the “best fit” to update likelihood information. Iteratively, the updated likelihood information will update the probability distribution of *γ*. Similar updating process is applied to all the parameters. The posterior samples of the parameter of interest could then be obtained via Markov Chain Monte Carlo (MCMC) calculations by various sampling algorithms, such as Gibbs sampling [52] or Metropolis-Hastings [53].

The selection of prior distributions for each parameter depends on the prior knowledge and interpretation of the parameter. For example, when the response variable is binary, i.e., *Y*_*i*_~*Bernoulli*(1, *p*_*i*_), the parameter *p*_*i*_ represents the probability of *Y*_*i*_ = 1. In this case, a beta distribution is usually considered as the prior distribution of *p*_*i*_, because the beta distribution is defined on the interval [0, 1] which matches the natural probability range (between 0 and 1). In the BMA method, parameter *γ* indicates which exposure vector is selected in likelihood calculation, and given a multinomial distribution with probability vector *π* = (*π*_1_,  … , *π*_*M*_) as its prior distribution. Multinomial distribution is a multivariate generalization of binomial distribution, which describes a trial with multiple possible outcomes. Since we have *M* sets of possibly true exposure vectors, it is appropriate to consider a multinomial distribution for *γ*. A Dirichlet distribution is often combined with a multinomial distribution to define the prior of the probability vector in multinomial distribution. In our case, each parameter in the probability vector *π* = (*π*_1_,  … , *π*_*M*_) represents the probability of selecting the corresponding exposure vector in likelihood calculation. For example, *π*_1_ = 0.6 indicates that the first exposure vector has 60% chance to be selected to update the likelihood. Therefore, a prior distribution of *Dirichlet*(1,  … , 1) for hyper-parameter vector *π* = (*π*_1_,  … , *π*_*M*_) is considered and it indicates that every exposure vector ${D}_r^{est}={\left[{D}_{1r}^{est},\dots, {D}_{Nr}^{est}\right]}^T$ has an equal a priori probability to be selected as the best fitting vector in the likelihood calculation. For additional details of BMA method see Kwon et al. [51].

Several radiation epidemiological studies have applied the BMA method to account for uncertainties in dose estimation [8, 9, 54]. For example, Land et al. (2015) examined the risk of radiation-related thyroid nodules in individuals who lived downwind from the Semipalatinsk Nuclear Test Site in Kazakhstan and accounted for complex uncertainties in dose estimation by using the BMA method [8]. Compared to conventional regression using a point “best estimate” dose [55], the BMA method increased the ERR per unit dose (ERR/Gy) estimate for the internal exposure, which was considered to have a large amount of shared uncertainties, by more than three times (see Table 2).

### Representative studies

Tables 2 and 3 present a selection of representative studies from radiation epidemiology and other environmental epidemiological studies, respectively. The presented studies applied at least one of the four methods to account for exposure estimation errors we reviewed above. Whenever possible, we looked for studies that used multiple statistical methods on same dataset.

In the majority of studies, risk estimates adjusted for exposure estimation uncertainties changed by +/− 100% compared to model without such adjustments, with the exception of two studies (Land et al. (2015) in Table 2 and Wang and Song (2016) in Table 3) that doubled/tripled the naïve risk estimates. Epidemiological textbooks state that random errors in exposure estimation lead to attenuation of exposure-response relationship. Thus, we expect that after accounting for exposure estimation uncertainties, risk estimates should increase. Accounting for Berkson error will usually lead to a wider confidence interval but would not bias risk estimates in linear models because Berkson error is usually caused by group averaging (i.e., *E*(*D*^*tr*^| *D*^*est*^) = *D*^*est*^) and is considered independent of estimated exposure values. However, in studies presented in Tables 2 and 3, the changes in risk estimates were not always away from the null. This could be due to complex error structures in different datasets or to different statistical methods applied. For example, BMA method works well when shared errors are substantial. However, it might “over-adjust” risk estimates if shared errors are small to only moderate in exposure estimation. Similarly, when shared errors are large, applying regression calibration or SIMEX could lead to “under-adjustment” of the uncertainties in exposure estimate.

## Discussion

In this paper, we provided a detailed description of four main methods to account for effects of uncertainties in exposures on exposure-response estimates used in radiation epidemiology (regression calibration, simulation-extrapolation (SIMEX), Monte Carlo maximum likelihood (MCML) and Bayesian model averaging (BMA)). Some of these methods have successfully been applied in several studies of environmental exposures (Table 3).

Regression calibration is easy to perform and works well when *E*(*D*^*tr*^| *D*^*est*^) can be approximated reasonably well (e.g., when validation data or data on an unbiased instrumental variable of exposure are available) or when a linear model is used for risk analysis. For example, linear ERR model is often used in radiation epidemiology to explore dose-response relationships and regression calibration works well for adjustment of risk estimates for uncertainties in exposure estimates. However, it is relatively weak for highly nonlinear models [3] or complex uncertainty structures [54]. For example, in radiation epidemiological studies, a complex uncertainty structure includes shared errors that usually cannot be ignored. However, in regression calibration, the individual exposure vector $\left({D}_{i1}^{est},\dots, {D}_{iM}^{est}\ \right)$ is treated as a vector of replicated estimates for *i*^*th*^ subject and its mean is used as a best estimate of true exposure in regression calibration. In such case, the correlation between subjects (i.e., the shared information) is not accounted for, even if the estimated exposure is obtained from the 2DMC procedure. In other epidemiological studies, such as nutritional studies, shared error is not considered as critical in exposure estimation. Obtaining data from validation studies or data on unbiased instrumental variables is relatively easy in these studies, which makes the calibration function *E*(*D*^*tr*^| *D*^*est*^) much easier to implement. Therefore, regression calibration method is a strong tool for these studies to correct for exposure estimation uncertainties.

Compared to regression calibration, SIMEX does not require an assumption about a distribution of the unknown true exposures and therefore would produce a relatively robust estimator [15]. Also, SIMEX is easy to perform because only a naïve estimator using estimated exposure values is used and no additional data are needed. However, SIMEX estimator can be affected by the variance of error term and the choice of extrapolation functions [37, 50]. We need to know the error variance or be able to estimate it precisely, or the results would not be accurate. SIMEX has the same weakness as the regression calibration method when a complex uncertainty structure is considered, because it also uses the individual exposure vector in the analysis.

In contrast to regression calibration and SIMEX, full-likelihood methods such as MCML and BMA use the possible true exposure vector for the entire cohort $\left({D}_{1r}^{est},\dots, {D}_{Nr}^{est}\ \right)\ \left(r=1,\dots, M\right)$ in exposure-response analyses, and therefore the shared information between subjects is preserved. Unlike regression calibration and SIMEX methods, which rely on the variance of the error term of exposure estimates, MCML and BMA methods use each vector of exposure estimates as a possible true exposure vector for the entire cohort. However, these methods are computationally intensive and must be applied based on 2DMC exposure estimates. Specifically, MCML estimation is based on values of likelihood on the profile likelihood function at specified grid points (e.g., 100 points) for parameter of interest for each exposure vector. Computational burdens will be large when the number of parameters of interest is more than two since choosing range and grid point is cumbersome and the number of likelihood evaluations will grow exponentially. Meanwhile, different choices of range and grid points for the likelihood evaluation would have an impact on the accuracy of point estimation (i.e., proximity to the true value) and confidence interval estimation. We might not obtain the accurate estimation results from inappropriate choices of range and sparse grid points.

When the shared uncertainties are relatively modest (e.g., [9, 54]), the full-likelihood methods are expected to work similarly to regression calibration. As demonstrated in Table 2, the regression calibration, MCML, and BMA methods had roughly similar results of reduced excess odds ratio per Gy (EOR/Gy) by 13, 2 and 23%, respectively, compared to the EOR/Gy from the models with no adjustment for dose uncertainties in studies of thyroid cancer after the Chornobyl accident [9]. The relatively small amount of shared errors is considered to be the cause of these modest effects from the application of adjustment methods in the exposure-response analyses. Unlike regression calibration and SIMEX methods, for which variance of exposure estimation errors is required, MCML and BMA methods require less information because each exposure estimate vector used in likelihood calculation is a possible true exposure vector for the entire cohort. However, if the shared uncertainties are substantial (e.g., same biased measuring device is applied to a group of people), the full likelihood methods such as MCML and BMA would perform better than regression calibration and SIMEX (see studies by Land et al. (2015) and Stayner et al. (2007), Table 2). The majority of the reviewed studies show relatively moderate changes (within 100%) in risk estimates after accounting for uncertainties in exposure estimates except for the two studies which doubled/tripled the naïve estimates [8, 56]. However, because the majority of risk estimates from studies of environmental exposures only show an excess of risk in exposed over unexposed of less than 100% (relative risks less than 2.0), the error in risk estimates of this magnitude is important. The risk estimates from analyses that do not account for uncertainties in exposure estimates could be significantly biased, and confidence in their accuracy overly optimistic. If analyses accounting for uncertainties in exposure estimates are not feasible, at least the potential effects of uncertain exposure estimates on final results should be discussed in environmental epidemiological studies when risk estimates are reported [5].

Other methods have been developed to account for uncertainties in exposure estimation in epidemiological studies at the stage of data analysis. Zhang et al. (2017) described a corrected confidence interval (CCI) approach to correct inflated variances of risk parameters estimated by the Poisson regression model due to uncertainties in the dosimetry system [57]. The CCI approximates an asymptotic distribution of parameter estimates in Poisson ERR model using multiple exposure vectors from the Monte Carlo dosimetry system. The CCI includes a variance-covariance matrix between multiple exposure vectors and mean exposure vector in the calculation of variances of parameters in the Poisson risk model. If exposure estimation uncertainty is large, then the corrected variances should be larger than the naïve variance estimates, which do not take account of exposure estimation uncertainties. Exposure-response analyses are performed with a mean exposure value of multiple exposure vectors using a regular Poisson ERR model to obtain an unbiased estimate of the risk parameter. Then, CCI is obtained by using corrected variances. The CCI is always wider than that of naïve approach due to the inflated variance estimate.

 The CCI approach has a big disadvantage when exposure estimation uncertainties are very large compared to MCML and BMA. When exposure estimation uncertainties are small or moderate, using a variance-covariance matrix between multiple exposure vectors and mean exposure vector reflects uncertainty, since each exposure vector has a very similar goodness-of-fit for the outcome. When exposure estimation uncertainty is large, the variance-covariance matrix between multiple exposure vectors and the mean exposure vector is excessively large and produces an unreasonably wide 95% confidence interval. In this situation, only a few exposure vectors provide a relatively strong goodness-of-fit while most others have a poor goodness-of-fit. Both MCML and BMA take account of this fact, and only a few exposure vectors contribute to the estimation of ERR and corresponding confidence interval. Using the variance-covariance matrix between multiple exposure vectors and the mean exposure vector as proposed by Zhang et al. (2017) does not incorporate this mechanism and thus produces an unnecessarily large variance for the corrected confidence interval.

Another method which has been used to account for uncertainties in exposure estimation at the stage of data analysis is a Multi-Model Inference (MMI) method, e.g. [58–60]. In order to avoid a biased result based on a single risk model, the MMI method combines risk estimates from multiple plausible exposure-response models by assigning a weight to each model. This method could provide a comprehensive evaluation of model uncertainties in risk estimates [5]. Conceptually, this method is similar to BMA and MCML in that uncertainty in the use of multiple realizations of possibly true model parameter values used to estimate individual exposure is similar to uncertainty in the use of multiple model structures or equations to estimate exposure.

## Conclusions

Although a single type of error may dominate in environmental epidemiological studies, uncertainties in exposure estimates for the entire cohort are often represented by more complex structures. Comprehensive consideration of potential error structures in the exposure estimates is important when developing an exposure estimation protocol because it can lead to improved exposure-response relationship by eliminating biases that can occur when uncertainties are ignored. If the exposure assessment is relatively simple and performed independently across individuals, unshared errors are more likely to be introduced. In such cases, using regression calibration and SIMEX methods with repeated estimates of exposure would work well to account for exposure estimation uncertainties in risk analyses. However, if the exposure assessment requires applying the same measurement device or using the same estimation parameters/models for a group of people, shared uncertainties are more likely to be introduced. In such cases, a more complicated exposure estimation method, i.e., 2DMC, needs to be considered. Although the 2DMC procedure was originally developed for radiation dose reconstruction, it could be easily used in other field of environmental epidemiology. Using exposure estimates from the 2DMC simulations, the MCML and BMA methods are able to account for exposure estimation uncertainties when shared errors are substantial. The methods reviewed in this paper are suitable to account for estimation errors in various situations of uncertain exposure estimates in environmental epidemiology. More analyses of uncertainties in exposure estimation should be conducted and the effects of uncertain exposure estimates on risk estimates should be discussed in environmental epidemiological studies when risk estimates are reported.

## Abbreviations

2DMC: Two-dimensional Monte Carlo
AIC: Akaike information criterion
BIC: Bayesian information criterion
BMA: Bayesian model averaging
CCI: Corrected confidence interval
EOR: Excess odds ratio
EPA: The U.S. Environmental Protection Agency
ERR: Excess relative risk
FFQ: Food frequency questionnaire
Gy: Gray
MCMC: Markov chain Monte Carlo
MCML: Monte Carlo maximum likelihood
MMI: Multi-model inference
NRC: National Research Council
PM: Particulate matter
SIMEX: Simulation-extrapolation
Sv: Sievert
